# Prognostic Value of Neutrophil-to-Lymphocyte Ratio and Vaccination for Negative Conversion Time of Nucleic Acid in Nonsevere COVID-19 Patients Infected by SARS-CoV-2 Omicron Variant

**DOI:** 10.1155/2023/9576855

**Published:** 2023-09-25

**Authors:** Kongbo Zhu, Shaolei Ma, Hui Chen, Jianfeng Xie, Dan Huang, Zhenghua Hou, Shanhu Qiu, Genshan Ma, Yingzi Huang

**Affiliations:** ^1^Department of Cardiology, Zhongda Hospital, School of Medicine, Southeast University, Nanjing, China; ^2^Department of Emergency and Critical Care Medicine, Zhongda Hospital, School of Medicine, Southeast University, Nanjing, China; ^3^Department of Critical Care Medicine, Zhongda Hospital, School of Medicine, Southeast University, Nanjing, China; ^4^Department of Psychosomatics and Psychiatry, Zhongda Hospital, School of Medicine, Southeast University, Nanjing, China; ^5^Department of General Practice, Zhongda Hospital, School of Medicine, Southeast University, Nanjing, China

## Abstract

SARS-CoV-2 Omicron variant is significantly different from all the previous variants and has rapidly replaced other variants as the dominant variant across the globe. An easily obtained, inexpensive, and rapid marker is needed to predict the negative conversion time (NCT) of nucleic acid in nonsevere COVID-19 patients infected by the Omicron variant. This retrospective study enrolled 226 patients infected by the Omicron variant between April 23, 2022, and May 16, 2022. The median age of the patients was 61 (interquartile range (IQR), 48–70) years, and 56.2% were male. 84 patients (37.2%) had at least one comorbidity, and 49 patients (21.7%) were classified into the moderate illness group. 145 patients (64.2%) received at least one dose of vaccine, in which 67 patients (29.6%) received a booster dose of vaccine. The median duration of NCT was 8 (IQR, 7–11) days. Univariate Cox analyses found that high NLR (>2.22), aged ≥65 years, vaccination, and moderate illness were significantly related to the NCT of nucleic acid. Multivariate Cox regression analysis showed that high NLR (NLR > 2.22, hazard ratio (HR):0.718, 95% CI: 0.534–0.964, *p* = 0.028) and vaccination (vaccinated ≥1 dose, HR: 1.536, 95% CI: 1.147–2.058, *p* = 0.004) were independently associated with NCT of nucleic acid. NLR is a rapid, simple, and useful prognostic factor for predicting NCT of nucleic acid in nonsevere COVID-19 patients with the Omicron variant. In addition, vaccination may also play a valuable role in predicting the NCT of nucleic acid.

## 1. Introduction

Since December 2019, coronavirus disease 2019 (COVID-19), caused by severe acute respiratory syndrome coronavirus 2 (SARS-CoV-2) had spread rapidly to many countries [1]. SARS-CoV-2 evolves through random mutations, and new mutations can potentially increase or decrease infectiousness and virulence. The Omicron variants of concern (VOC) (Phylogenetic Assignment of Named Global Outbreak (Pango) lineage designation B.1.1.529) of SARS-CoV-2 emerged in South Africa in November 2021. It is more transmissible than other variants [2]. Therefore, the Omicron variant rapidly replaced previous variants as the dominant variant across the globe [3]. In late February 2022, a wave of SARS-CoV-2 Omicron variant infection rapidly appeared in Shanghai, China. Phylogenetic features of SARS-CoV-2 viral genomes from 129 patients in Shanghai indicated that all of the new viral genomes were clustered into the SARS-CoV-2 BA.2.2 sublineage.

The detection of SARS-CoV-2 nucleic acid by reverse transcription polymerase chain reaction (RT-PCR) is still the golden standard for the diagnosis of COVID-19, and it is also of great significance for determining discharge and isolation. The clinical prediction of negative conversion is critical for the proper retesting time, preventing medical waste from repeated nucleic acid tests and unnecessarily prolonged quarantine. The negative conversion time (NCT) of nucleic acid is also important in terms of viral transmission. The Omicron variant spreads much faster than the other variants, but the mean length of hospital stays was shorter [4, 5]. However, there is limited data regarding the potential predictors of NCT in COVID-19 patients with the SARS-CoV-2 Omicron variant. Therefore, we need an easily obtained, inexpensive, and rapid marker to predict the NCT of nucleic acid in nonsevere COVID-19 patients with the SARS-CoV-2 Omicron variant.

Inflammation can be caused by infectious diseases, and there is a dysregulated immune response in patients with COVID-19 [6]. As an independent predictor of disease deterioration and mortality in COVID-19 patients [7–14], neutrophil-to-lymphocyte ratio (NLR) is closely related to the pathophysiology of COVID-19. The NLR is calculated as the absolute neutrophil count divided by the lymphocyte count. It is a reliable predictor of COVID-19 progression and can differentiate between mild/moderate and severe/critical groups. Higher NLR is associated with higher mortality. Neutrophil to CD4^+^ lymphocyte ratio and lower levels of CD3^+^CD4^+^ lymphocytes were also useful blood markers in previous studies [15, 16]. However, the CD4^+^ lymphocyte test needs flow cytometry which is inconvenient to detect during a large-scale epidemic. As a simple marker of inflammation, NLR can be easily obtained from routine blood tests. But the Omicron variant evolves towards being less virulent and is very different from the previous variants [17]. The prognostic value of NLR as a predictor of the NCT of the nucleic acid of COVID-19 patients infected by Omicron variant Omicron is unknown.

COVID-19, caused by the SARS-CoV-2 Omicron variant, has become very different from that in the early outbreaks in the world. Although the Omicron variant evolves towards less pathogenic, the mortality rate of immunocompromised patients infected with Omicron variant is significantly higher than that of nonimmunocompromised patients [18]. Vaccinated immunocompromised patients had a poor humoral response. A higher rate of mortality has also been reported in unvaccinated and not fully or effectively vaccinated elderly people [19]. Although the vaccines have been widely covered, vaccination rates for older people remain low. The coverage of the elderly still needs to be improved. The strict and comprehensive pandemic control strategies can reduce the number of elderly people infected by the Omicron variant so that the mortality rate can be minimized and we can buy time for full vaccination coverage. Like other RNA viruses, SARS-CoV-2 is evolving through random mutations, and new mutations can potentially increase or decrease virulence and infectiousness. Moreover, mutations can increase the ability of the virus to evade adaptive immune responses from past SARS-CoV-2 infections or vaccinations. Vaccination rates were not included in previous studies on the NCT of nucleic acid, and the impact of vaccines on the NCT of nucleic acid is still unknown.

Decisions on infection prevention continue to be broadly guided by strategies based on the NCT of nucleic acid. Therefore, further investigation on the duration of NCT of nucleic acid and factors associated with prolonged negative conversion conducted among larger populations may help to improve the clinical management of COVID-19 patients infected by the SARS-CoV-2 Omicron variant. In the current study, we aimed to assess the prognostic value of NLR and identify the clinical characteristics, including vaccination, that influence the NCT of nucleic acid in nonsevere COVID-19 patients infected by the SARS-CoV-2 Omicron variant.

## 2. Materials and Methods

### 2.1. Study Design and Participants

This retrospective cohort study enrolled patients with confirmed COVID-19 hospitalized in Lingang shelter hospital, Shanghai, China, between April 23, 2022, and May 16, 2022. Apart from the function of isolating COVID-19 patients, the Lingang shelter hospital provided basic medical services for nonsevere COVID-19 patients, including asymptomatic infection, mild illness, and moderate illness individuals.

COVID-19 patients above the age of 18 years with complete clinical data and blood tests in which samples were collected prior to negative conversion of nucleic acid were included. Cases that met one of the following criteria were excluded: (1) severe illness individuals; (2) critical illness individuals; (3) past or present medical history of chronic illness affecting NLR values: autoimmune diseases, malignancies under treatment, inflammatory chronic diseases, chronic hematological disorder, gastrointestinal bleeding, recent acute myocardial damage, recent surgical procedures, HIV infection, and cirrhosis; (4) pregnant and lactating women; and (5) previous pharmacological treatment affecting NLR values such as corticosteroids.

Referring to the WHO criteria, the severity of the disease is classified according to the following criteria. Asymptomatic infection: individuals who test positive for SARS-CoV-2 using a nucleic acid amplification test but who have no symptoms that are consistent with COVID-19. Mild illness: individuals who have any of the various signs and symptoms of COVID-19 but do not have shortness of breath, dyspnea, or abnormal chest imaging. *Moderate Illness*: individuals who show evidence of lower respiratory disease during CT examinations and who have an oxygen saturation (SpO_2_) ≥94% in room air at sea level. *Severe Illness*: individuals who have SpO_2_ <94% in room air at sea level, a ratio of arterial partial pressure of oxygen to fraction of inspired oxygen (PaO_2_/FiO_2_) <300 mmHg, a respiratory rate >30 breaths/min, or lung infiltrates >50%. *Critical Illness*: individuals who have respiratory failure, septic shock, and/or multiple organ dysfunction.

### 2.2. Data Collection

Patients' health information stored in the electronic medical records system was collected. We collected available clinical variables, including demographic characteristics, vaccination status, comorbidities, and laboratory tests. Comorbidities included hypertension, diabetes, cerebro-cardiovascular diseases, chronic respiratory diseases (asthma, chronic obstructive pulmonary disease, or interstitial lung disease), and chronic kidney disease. Laboratory tests comprised white leukocyte count, neutrophil count, lymphocyte count, monocyte count, platelet count, hemoglobin, aspartate aminotransferase (AST), alanine aminotransferase (ALT), serum creatinine, and D-dimer. The derived hematological indicator was NLR. The blood test data collected were the results of the first examination after admission before the negative conversion of nucleic acid tests.

### 2.3. NCT of Nucleic Acid

Strict pandemic control strategies were taken in Shanghai, and nucleic acid detections were carried out regularly among Shanghai residents. If the nucleic acid test was positive, they would be transferred to the shelter hospitals or designated tertiary hospitals. Nasopharyngeal swab specimens were collected from each patient every day during hospitalization. RNA was extracted from the samples and then underwent RT-PCR by DIAN DIAGNOSTICS Group Co., Ltd., Shanghai, China, which has a laboratory that provided nucleic acid testing and analysis for Lingang shelter hospital. A cycle threshold (*Ct*) value (N gene and ORF gene) of 40 or more was considered a negative test. The standard of negative conversion was two successive negative nucleic acid tests at minimum 24-hour sampling intervals. The first nucleic acid negative date of two consecutive nucleic acid negative dates after admission was defined as the negative date of nucleic acid. The primary outcome of this study is the NCT of nucleic acid, which was calculated as the number of days between the date of nucleic acid positive in community screening before admission and the negative date of nucleic acid after admission [20, 21].

### 2.4. Ethical Considerations

This study was authorized by the Ethics Commission for Clinical Research of Zhongda Hospital, affiliated with Southeast University (approval number: 2022ZDSYLL170-P01; approval date: May 31, 2022). Informed consent was waived due to the nature of the retrospective study. The study was performed under the principles stated in the Declaration of Helsinki, and the confidentiality of patients was guaranteed.

### 2.5. Statistical Analysis

Categorical variables are presented as the number and percentage of the total. Continuous variables are shown as the median (interquartile range (IQR)). The optimal cut-off value for NLR was calculated by X-tile software (Yale University, New Haven, CT, USA) [22]. As time-to-event data, NCT of nucleic acid was the outcome measure and presented with Kaplan–Meier curves. The log-rank test was used for the comparison. To detect the independent predictors of NCT of nucleic acid, univariate and multivariate Cox regression analyses were performed and reported as the hazard ratio (HR) and 95% confidence interval (CI). Data were analyzed using SPSS 25.0 software (SPSS Inc., Chicago, IL). A two-tailed *p* value <0.05 was considered statistically significant.

## 3. Results

235 patients who were above the age of 18 years with complete clinical data and blood tests satisfied the inclusion criteria. Among them, 4 patients were excluded from the study for severe illness of COVID-19. Furthermore, 5 patients were excluded because of autoimmune diseases (3 patients) and malignancies under treatment (2 patients). The study finally included 226 patients with nonsevere COVID-19 infections.

**Figure 1:**
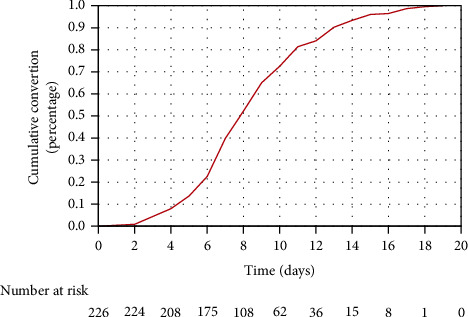
Negative conversion curves estimating the cumulative probability of negative conversion of nucleic acid in total nonsevere COVID-19 patients infected by the SARS-CoV-2 Omicron variant.

As shown in Table 1, the median age of the patients was 61 years, and 95 patients (42.0%) were elderly (≥65 years). 127 patients (56.2%) were male, and more than one-third of the patients (37.2%, *n* = 84) had at least one comorbidity, including hypertension (31.0%, *n* = 70), diabetes (13.3%, *n* = 30), cerebro-cardiovascular diseases (2.7%, *n* = 6), chronic respiratory disease (2.2%, *n* = 5), and chronic kidney disease (3.1%, *n* = 7). 145 patients (64.2%) received at least one dose of inactivated SARS-CoV-2 vaccine (vero cell), in which 67 patients (29.6%) received a third (booster) dose of inactivated vaccine. Only 6 patients (2.7%) received one dose of the inactivated vaccine, and 72 patients (31.9%) got two doses of the inactivated vaccine. 49 patients (21.7%) were classified into the moderate illness group because the CT scans showed pneumonia. Lymphopenia (<1.1 × 10^9^ cells/L) was found in 64 patients (28.3%), and 10 patients (4.4%) had neutrophilia (>6.3 × 10^9^ cells/L). A low eosinophil level (<0.02 × 10^9^ cells/L) was present in 38 patients (16.8%), and monocytosis (>0.6 × 10^9^ cells/L) was observed in 48 patients (21.2%). The median NLR was 2.06 (IQR, 1.47–3.04) (Table 1). The range of NCT of nucleic acid in this study was from 2 to 19 days, and the median duration was 8 (IQR, 7–11) days (Figure 1).

**Figure 2:**
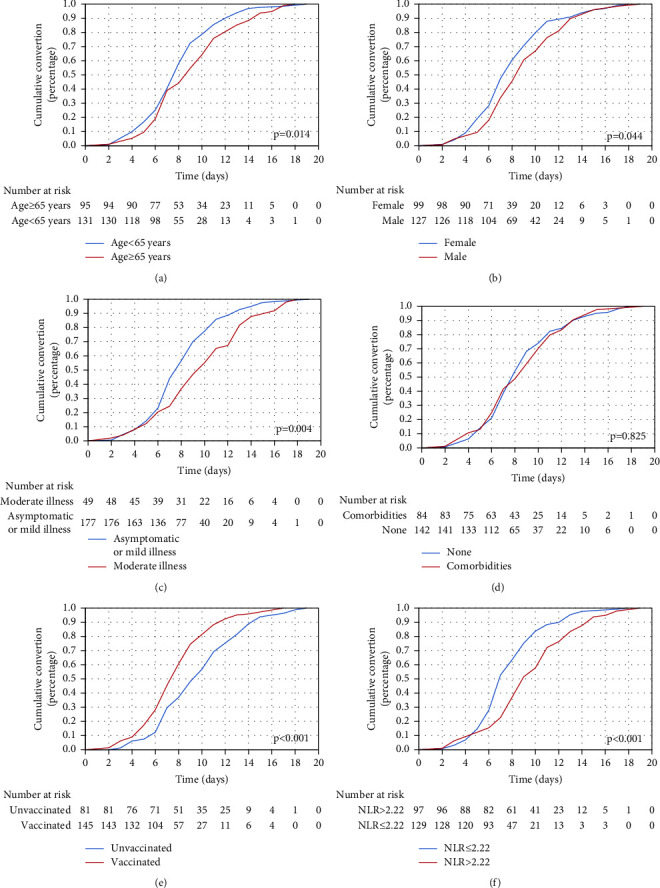
Negative conversion curves estimating the cumulative probability of negative conversion of nucleic acid in nonsevere COVID-19 patients infected by the SARS-CoV-2 Omicron variant after stratifying by (a) age (age ≥ 65 years versus age<65 years), (b) gender (male versus female), (c) disease severity (moderate illness versus asymptomatic or mild illness), (d) comorbidities (comorbidities versus none), (e) vaccination (vaccinated ≥1 dose versus unvaccinated), and (f) NLR (NLR > 2.22 versus NLR ≤ 2.22).

Kaplan–Meier analysis results on differences in the NCT of nucleic acid based on clinical characteristics are shown in Figure 2. Elderly patients (≥65 years) had remarkably higher risks of delayed NCT of nucleic acid than those <65 years (log-rank: *p* = 0.014, *χ*^2^ = 6.062, Figure 2(a)). Male patients had extremely longer NCT of nucleic acid than female patients (log-rank: *p* = 0.044, *χ*^2^ = 4.038, Figure 2(b)). NCT in moderately ill patients was significantly increased (log-rank: *p* = 0.004, *χ*^2^ = 8.339, Figure 2(c)), while that in vaccinated patients was significantly reduced (log-rank: *p* < 0.001, *χ*^2^ = 17.776, Figure 2(e)). Comorbidities did not affect the NCT of nucleic acid nevertheless (log-rank: *p* = 0.825, *χ*^2^ = 0.049, Figure 2(d)). To find the ideal cut-off value of NLR, X-tile software was used. Finally, the optimal cut-off point of NLR was 2.22 in this study (Supplementary Figure 1). Meanwhile, patients in the high NLR group had remarkably higher risks of delayed NCT of nucleic acid than those in the low NLR group (log-rank: *p* < 0.001, *χ*^2^ = 17.427, Figure 2(f)).

We evaluated the effect of each factor on the NCT of nucleic acid using the Cox regression analysis for univariate analysis.  Table 2 summarized the results of univariate analyses. High NLR (>2.22, HR: 0.604, 95% CI: 0.461–0.790, *p* < 0.001), aged (≥65 years, HR: 0.743, 95% CI: 0.568–0.971, *p* = 0.030), vaccination (≥1 dose, HR: 1.699, 95% CI: 1.283–2.25, *p* < 0.001), and moderate illness (HR: 0.662, 95% CI: 0.480–0.912, *p* = 0.012) were significantly related to NCT of nucleic acid. The male (HR: 0.787, 95% CI: 0.604–1.025, *p* = 0.075), low eosinophil level (<0.02 × 10^9^ cells/L, HR: 0.847, 95% CI: 0.596–1.202, *p* = 0.351), monocytosis (>0.6 × 10^9^ cells/L, HR: 0.842, 95% CI: 0.611–1.159, *p* = 0.291), and comorbidities (HR: 0.973, 95% CI: 0.742–1.277, *p* = 0.845) did not have an impact on the NCT of nucleic acid.

Multivariate Cox regression was then performed to find independent factors associated with the NCT of nucleic acid. After adjusting for age, sex, comorbidities, moderate illness, low eosinophil level, and monocytosis, high NLR (NLR > 2.22, HR: 0.718, 95% CI: 0.534–0.964, *p* = 0.028) and vaccination (vaccinated ≥ 1 dose, HR: 1.536, 95% CI: 1.147–2.058, *p* = 0.004) were independently associated with NCT of nucleic acid (Table 2), suggesting that NLR > 2.22 would delay NCT of nucleic acid in COVID-19 patients and vaccination (vaccinated ≥ 1 dose) could reduce the NCT of nucleic acid.

## 4. Discussion

Increased NCT of nucleic acid is an independent risk factor for prolonged hospitalizations and quarantine [23]. Several studies have evaluated the predictors of NCT infected by other SARS-CoV-2 variants before Omicron. The Omicron variant is different from the previous variant, and there is limited data regarding the potential predictors of negative conversion in the Omicron variant, particularly in high-risk patients. In our study, univariate analysis revealed that clinical characteristics such as the aged (age ≥ 65 years), moderate illness, vaccination, and high NLR were significantly related to the NCT of nucleic acid. Comorbidities were not statistically significant. Multivariate Cox regression showed that high NLR and vaccination were independently associated with the NCT of nucleic acid. In this study, we reported that NLR might be a simple and useful prognostic factor in the prediction of NCT of nucleic acid in nonsevere COVID-19 patients infected by the SARS-CoV-2 Omicron variant. In addition, we also found that vaccination played a valuable role in predicting the NCT of nucleic acid.

42.0% of the patients in this study were elderly, and more than one-third of the patients (37.2%) had at least one comorbidity. In addition, 49 patients (21.7%) were diagnosed with pneumonia by CT scans and classified into the moderate illness group. Patients enrolled in this study seem to be more likely to be in the high-risk group. In our cohort, the median duration of NCT of nucleic acid was 8 days, ranging from 2 to 19 days. This finding is markedly shorter compared with previous studies focused on other mutant strains [24–29], which reported that the mean NCT of nucleic acid was longer than 10 days. This may be attributed to the weaker pathogenicity of the Omicron variant. The NCT of nucleic acid in this study was similar to the result in another study on Omicron [30], in which the median time was 6 days. More recently, public health guidelines have recommended a shorter period of strict isolation from the onset of symptoms or after the initial positive test. Interestingly, there is still a proportion of patients with NCT of nucleic acid longer than 10 days, and this group of patients, if isolated for a short period, could become a source of infection and thus infect a larger population.

Symptoms such as chest tightness, fever, respiratory symptoms, and digestive symptoms have showed a good performance in predicting the NCT of nucleic acid [31–33]. But Omicron has an increasing portion of patients with asymptomatic infection; difficulties exist in studying the value of symptoms. A longer NCT of nucleic acid has been variously associated with advanced age, disease severity, delayed hospital admission, and comorbidities [25, 34, 35]. It is worth noting that age or comorbidities did not have a significant relationship with prolonged NCT of nucleic acid during multivariate Cox regression analysis in our study, which is consistent with the previous study [33]. Patients with certain underlying comorbidities are at higher risk of progressing to severe COVID-19 [31]. However, the findings of our study suggest that the importance of such underlying comorbidities may not be significant in the duration of disease, which is one of the main treatment outcomes in the group of nonsevere COVID-19 patients. Even though our study population was predominantly elderly, underlying comorbidities did not have a relationship with the NCT of nucleic acid in univariate and multivariate analyses. This finding could be characteristics of asymptomatic or mildly symptomatic patients of COVID-19 infected by the SARS-CoV-2 Omicron variant. However, the results of this study cannot be applied to all patients with COVID-19 infected by the SARS-CoV-2 Omicron variant of varying severity.

We demonstrated that NLR is an independent factor associated with NCT of nucleic acid, and high NLR (>2.22) would predict the delayed negative conversion in nonsevere COVID-19 patients with Omicron variant. NLR is an indicator of the systematic inflammatory response and has been widely investigated as a reliable predictor of COVID-19 progression [36]. SARS-CoV-2-triggered inflammation increased NLR, and elevated NLR promoted COVID-19 progression. NLR is significantly higher in patients with severe COVID-19 [37, 38]. Finally, multivariate regression analysis showed that high NLR was independently associated with the NCT of nucleic acid in our study. As expected, the cut-off value of NLR in this study is lower than that for identifying severe/critical patients [39]. NLR is a rapid, widely available, inexpensive marker which can be easily obtained from a simple blood test, and it can be widely used in the management of high-risk Omicron infection patients during the pandemic.

In addition, this study also found that vaccination was an independent predictor of NCT of nucleic acid, and patients in the unvaccinated group had remarkably higher risks of delayed NCT of nucleic acid than those in the vaccinated group. The Omicron variant may evade immunity from previous vaccines or infections more extensively than any other variant, making existing vaccines less effective against the variant [40–42]. But vaccinated people are likely to have a much lower risk of severe disease from Omicron infection. The boosters of the COVID-19 vaccination are less effective against symptomatic Omicron infection, but they can provide strong protection against COVID-19-related hospitalization and death [43–45]. In this study, vaccines help shorten the NCT of nucleic acid in nonsevere COVID-19 patients with Omicron infection. However, only 64.2% of the patients in this study were vaccinated, and 29.6% received a booster dose of the vaccine. The possible reason for low vaccine coverage in our study is that patients are older and have more underlying diseases. These proportions are consistent with those of the whole society in Shanghai. Although overall vaccination coverage now exceeds 90%, vaccination coverage has remained low in elder adults [46]. The next challenge is to take steps to improve vaccine coverage for all people, the elder and vulnerable people in particular.

This study has several limitations. As a retrospective study, the study only involved nonsevere COVID-19 cases, so the conclusions cannot be employed for severe and critical Omicron cases. Undoubtedly, NLR is a traditional marker, but it is cheap, convenient, and maybe a better choice. Limited by the sample size, the distinctions between different doses of vaccines were not discussed, and large clinical studies are needed to confirm the protective effect of different doses and types of vaccines on NCT of nucleic acid.

## 5. Conclusions

In summary, the current study shows that NLR may be a rapid, simple, and useful prognostic factor for predicting NCT of nucleic acid in nonsevere COVID-19 patients with the SARS-CoV-2 Omicron variant, and it can be used extensively in the management of Omicron infection. In addition, this study also suggests that vaccination is an independent predictor of NCT of nucleic acid in nonsevere COVID-19 patients with Omicron infection. Measures should be to taken to improve vaccine coverage of the elderly and vulnerable people.

## Figures and Tables

**Table 1:** Clinical characteristics and laboratory findings of nonsevere COVID-19 patients infected by the SARS-CoV-2 Omicron variant.

Variables	Total (*N* = 226)
Age (years)	61 (48–70)
Age ≥ 65 years	95 (42.0%)
Male	127 (56.2%)
Comorbidities ≥ 1	84 (37.2%)
Hypertension	70 (31.0%)
Diabetes	30 (13.3%)
Cerebro-cardiovascular diseases	6 (2.7%)
Chronic respiratory diseases	5 (2.2%)
Chronic kidney disease	7 (3.1%)
Vaccinated ≥ 1 dose	145 (64.2%)
1 dose	6 (2.7%)
2 doses	72 (31.9%)
3 doses	67 (29.6%)
Moderate illness	49 (21.7%)
Leukocyte count, ×10^9^ cells/L	5.38 (4.23–6.45)
Lymphopenia (<1.1 × 10^9^ cells/L)	64 (28.3%)
Neutrophilia (>6.3 × 10^9^ cells/L)	10 (4.4%)
Platelet count, ×10^9^ cells/L	194 (157–244)
Hemoglobin, g/L	133 (119–146)
Low eosinophil level (<0.02 × 10^9^ cells/L)	38 (16.8%)
Monocytosis (>0.6 × 10^9^ cells/L)	48 (21.2%)
ALT, U/L	19 (13–27)
AST, U/L	22 (18–27)
Serum creatinine, umol/L	69 (58–78)
D-dimer, *μ*g/mL	0.44 (0.32–0.65)
NLR	2.06 (1.47–3.04)

^*∗*^Data are presented as median (interquartile range (IQR), 25%–75%) for continuous variables and as number with percentage for categorical variables. ^*∗*^ALT: alanine aminotransferase; ^*∗*^AST: aspartate aminotransferase; ^*∗*^NLR: neutrophil-to-lymphocyte ratio.

**Table 2:** Univariable and multivariate Cox regression analyses for risk factors associated with the NCT of nucleic acid in nonsevere COVID-19 patients infected by the SARS-CoV-2 Omicron variant.

Variables	Univariate analysis		Multivariate analysis	
	HR (95% CI)	*p* value	HR (95% CI)	*p* value
Aged (≥65 years)	0.743 (0.568–0.971)	**0.030**	0.812 (0.610–1.079)	0.151
Male	0.787 (0.604–1.025)	0.075	0.826 (0.621–1.098)	0.187
Comorbidities ≥ 1	0.973 (0.742–1.277)	0.845	1.090 (0.823–1.443)	0.548
Vaccinated ≥ 1 dose	1.699 (1.283–2.250)	**<0.001**	1.536 (1.147–2.058)	**0.004**
Moderate illness	0.662 (0.480–0.912)	**0.012**	0.831 (0.590–1.171)	0.290
Low eosinophil level (<0.02 × 10^9^ cells/L)	0.847 (0.596–1.202)	0.351	0.910 (0.634–1.307)	0.611
Monocytosis (>0.6 × 10^9^ cells/L)	0.842 (0.611–1.159)	0.291	1.016 (0.719–1.435)	0.929
NLR > 2.22	0.604 (0.461–0.790)	**<0.001**	0.718 (0.534–0.964)	**0.028**

^*∗*^HR: hazard ratio; ^*∗*^CI: confidence interval; ^*∗*^NLR: neutrophil-to-lymphocyte ratio; ^*∗*^NCT: negative conversion time. Bold value: *p* < 0.05.

## Data Availability

The data that support the findings of this study are available from the corresponding author upon reasonable request.

## Supplementary Materials

Supplementary Materials Supplementary Figure 1: the optimal cut-off value for NLR was calculated by X-tile software.

## References

[1] Li Q., Guan X., Wu P. (2020). Early transmission dynamics in wuhan, China, of novel coronavirus-infected pneumonia. *New England Journal of Medicine*.

[2] Balint G., Voros-Horvath B., Szechenyi A. (2022). Omicron: increased transmissibility and decreased pathogenicity. *Signal Transduction and Targeted Therapy*.

[3] Viana R., Moyo S., Amoako D. G. (2022). Rapid epidemic expansion of the SARS-CoV-2 Omicron variant in southern Africa. *Nature*.

[4] Maslo C., Friedland R., Toubkin M. (2022). Characteristics and outcomes of hospitalized patients in South Africa during the COVID-19 omicron wave compared with previous waves. *JAMA*.

[5] Wolter N., Jassat W., Walaza S. (2022). Early assessment of the clinical severity of the SARS-CoV-2 omicron variant in South Africa: a data linkage study. *The Lancet*.

[6] Qin C., Zhou L., Hu Z. (2020). Dysregulation of immune response in patients with coronavirus 2019 (COVID-19) in wuhan, China. *Clinical Infectious Diseases*.

[7] Seyit M., Avci E., Nar R. (2021). Neutrophil to lymphocyte ratio, lymphocyte to monocyte ratio and platelet to lymphocyte ratio to predict the severity of COVID-19. *The American Journal of Emergency Medicine*.

[8] Yu G. Q., Zhang Q., Wang R. C., Jiang S. Q. (2021). Predictive value of neutrophil-to-lymphocyte ratio and other inflammatory indicators in estimating clinical severity of coronavirus disease. *World Journal of Emergency Medicine*.

[9] Kong M., Zhang H., Cao X., Mao X., Lu Z. (2020). Higher level of neutrophil-to-lymphocyte is associated with severe COVID-19. *Epidemiology and Infection*.

[10] Yan X., Li F., Wang X. (2020). Neutrophil to lymphocyte ratio as prognostic and predictive factor in patients with coronavirus disease 2019: a retrospective cross-sectional study. *Journal of Medical Virology*.

[11] Regolo M., Vaccaro M., Sorce A. (2022). Neutrophil-to-Lymphocyte ratio (NLR) is a promising predictor of mortality and admission to intensive care unit of COVID-19 patients. *Journal of Clinical Medicine*.

[12] Liu J., Liu Y., Xiang P. (2020). Neutrophil-to-lymphocyte ratio predicts critical illness patients with 2019 coronavirus disease in the early stage. *Journal of Translational Medicine*.

[13] Wang Q., Cheng J., Shang J. (2021). Clinical value of laboratory indicators for predicting disease progression and death in patients with COVID-19: a retrospective cohort study. *BMJ Open*.

[14] Vafadar Moradi E., Teimouri A., Rezaee R. (2021). Increased age, neutrophil-to-lymphocyte ratio (NLR) and white blood cells count are associated with higher COVID-19 mortality. *The American Journal of Emergency Medicine*.

[15] Mo P., Deng L., Liu X. (2020). Risk factors for delayed negative conversion of SARS-CoV-2 in patients with COVID-19 pneumonia: a retrospective cohort study. *Epidemiology and Infection*.

[16] Wang H., Zhang Y., Mo P. (2020). Neutrophil to CD4+ lymphocyte ratio as a potential biomarker in predicting virus negative conversion time in COVID-19. *International Immunopharmacology*.

[17] Krutikov M., Stirrup O., Nacer-Laidi H. (2022). Outcomes of SARS-CoV-2 omicron infection in residents of long-term care facilities in England (VIVALDI): a prospective, cohort study. *The Lancet Healthy Longevity*.

[18] de Prost N., Audureau E., Heming N. (2022). Clinical phenotypes and outcomes associated with SARS-CoV-2 variant Omicron in critically ill French patients with COVID-19. *Nature Communications*.

[19] Cheung P. H., Chan C., Jin D. (2022). Lessons learned from the fifth wave of COVID-19 in Hong Kong in early 2022. *Emerging Microbes & Infections*.

[20] Hu X., Xing Y., Jia J. (2020). Factors associated with negative conversion of viral RNA in patients hospitalized with COVID-19. *The Science of the Total Environment*.

[21] Benoni R., Campagna I., Panunzi S. (2021). Estimating COVID-19 recovery time in a cohort of Italian healthcare workers who underwent surveillance swab testing. *Public Health*.

[22] Camp R. L., Dolled-Filhart M., Rimm D. L. (2004). X-tile: a new bio-informatics tool for biomarker assessment and outcome-based cut-point optimization. *Clinical Cancer Research*.

[23] Lin P., Chen W., Huang H. (2021). Delayed discharge is associated with higher complement C3 levels and a longer nucleic acid-negative conversion time in patients with COVID-19. *Scientific Reports*.

[24] Li N., Wang X., Lv T. (2020). Prolonged SARS-CoV-2 RNA shedding: not a rare phenomenon. *Journal of Medical Virology*.

[25] Xu K., Chen Y., Yuan J. (2020). Factors associated with prolonged viral RNA shedding in patients with coronavirus disease 2019 (COVID-19). *Clinical Infectious Diseases*.

[26] Zhou F., Yu T., Du R. (2020). Clinical course and risk factors for mortality of adult inpatients with COVID-19 in Wuhan, China: a retrospective cohort study. *The Lancet*.

[27] Qi L., Yang Y., Jiang D. (2020). Factors associated with the duration of viral shedding in adults with COVID-19 outside of Wuhan, China: a retrospective cohort study. *International Journal of Infectious Diseases*.

[28] Zhou B., She J., Wang Y., Ma X. (2020). Duration of viral shedding of discharged patients with severe COVID-19. *Clinical Infectious Diseases*.

[29] Cevik M., Tate M., Lloyd O. (2021). SARS-CoV-2, SARS-CoV, and MERS-CoV viral load dynamics, duration of viral shedding, and infectiousness: a systematic review and meta-analysis. *The Lancet Microbe*.

[30] Boucau J., Marino C., Regan J. (2022). Duration of viable virus shedding in SARS-CoV-2 omicron variant infection. *medRxiv*.

[31] Bennett S., Tafuro J., Mayer J. (2021). Clinical features and outcomes of adults with coronavirus disease 2019: a systematic review and pooled analysis of the literature. *International Journal of Clinical Practice*.

[32] Yang Y., Hu X., Xiong L. (2021). Clinical characteristics of hospitalized mild/moderate COVID-19 patients with a prolonged negative conversion time of SARS-CoV-2 nucleic acid detection. *BMC Infectious Diseases*.

[33] Lee Y. H., Hong C. M., Lee T. H. (2022). Factors associated with prolonged viral detection in asymptomatic and mildly symptomatic patients with SARS-CoV-2 infection. *J Infect Dev Ctries*.

[34] Mondi A., Lorenzini P., Castilletti C. (2021). Risk and predictive factors of prolonged viral RNA shedding in upper respiratory specimens in a large cohort of COVID-19 patients admitted to an Italian reference hospital. *International Journal of Infectious Diseases*.

[35] Hirai N., Nishioka Y., Sekine T. (2021). Factors associated with viral clearance periods from patients with COVID-19: a retrospective observational cohort study. *Journal of Infection and Chemotherapy*.

[36] Ma A., Cheng J., Yang J. (2020). Neutrophil-to-lymphocyte ratio as a predictive biomarker for moderate-severe ARDS in severe COVID-19 patients. *Critical Care*.

[37] La Torre G., Marte M., Massetti A. P. (2022). The neutrophil/lymphocyte ratio as a prognostic factor in COVID-19 patients: a case-control study. *European Review for Medical and Pharmacological Sciences*.

[38] Fors M., Ballaz S., Ramirez H. (2022). Sex-dependent performance of the neutrophil-to-lymphocyte, monocyte-to-lymphocyte, platelet-to-lymphocyte and mean platelet volume-to-platelet ratios in discriminating COVID-19 severity. *Front Cardiovasc Med.*.

[39] Yildiz H., Castanares-Zapatero D., Pierman G. (2021). Validation of neutrophil-to-lymphocyte ratio cut-off value associated with high in-hospital mortality in COVID-19 patients. *International Journal of General Medicine*.

[40] Zhang X., Wu S., Wu B. (2021). SARS-CoV-2 Omicron strain exhibits potent capabilities for immune evasion and viral entrance. *Signal Transduction and Targeted Therapy*.

[41] Andrews N., Stowe J., Kirsebom F. (2022). Covid-19 vaccine effectiveness against the omicron (B.1.1.529) variant. *New England Journal of Medicine*.

[42] Altarawneh H. N., Chemaitelly H., Ayoub H. H. (2022). Effects of previous infection and vaccination on symptomatic omicron infections. *New England Journal of Medicine*.

[43] Abu-Raddad L. J., Chemaitelly H., Ayoub H. H. (2022). Effect of mRNA vaccine boosters against SARS-CoV-2 omicron infection in Qatar. *New England Journal of Medicine*.

[44] Accorsi E. K., Britton A., Fleming-Dutra K. E. (2022). Association between 3 doses of mRNA COVID-19 vaccine and symptomatic infection caused by the SARS-CoV-2 omicron and delta variants. *JAMA*.

[45] Bjork J., Bonander C., Moghaddassi M. (2022). COVID-19 vaccine effectiveness against severe disease from SARS-CoV-2 Omicron BA.1 and BA.2 subvariants surveillance results from southern Sweden, December 2021 to March 2022. *Euro Surveillance*.

[46] Zhang X., Zhang W., Chen S. (2022). Shanghai’s life-saving efforts against the current omicron wave of the COVID-19 pandemic. *The Lancet*.

