# Supplementary figures and images for: Prognostic Value of Neutrophil-to-Lymphocyte Ratio and Vaccination for Negative Conversion Time of Nucleic Acid in Nonsevere COVID-19 Patients Infected by SARS-CoV-2 Omicron Variant
Source: Int J Clin Pract. 2023 Sep 25;2023:9576855. doi: 10.1155/2023/9576855 (PMC10545465; doi:10.1155/2023/9576855)

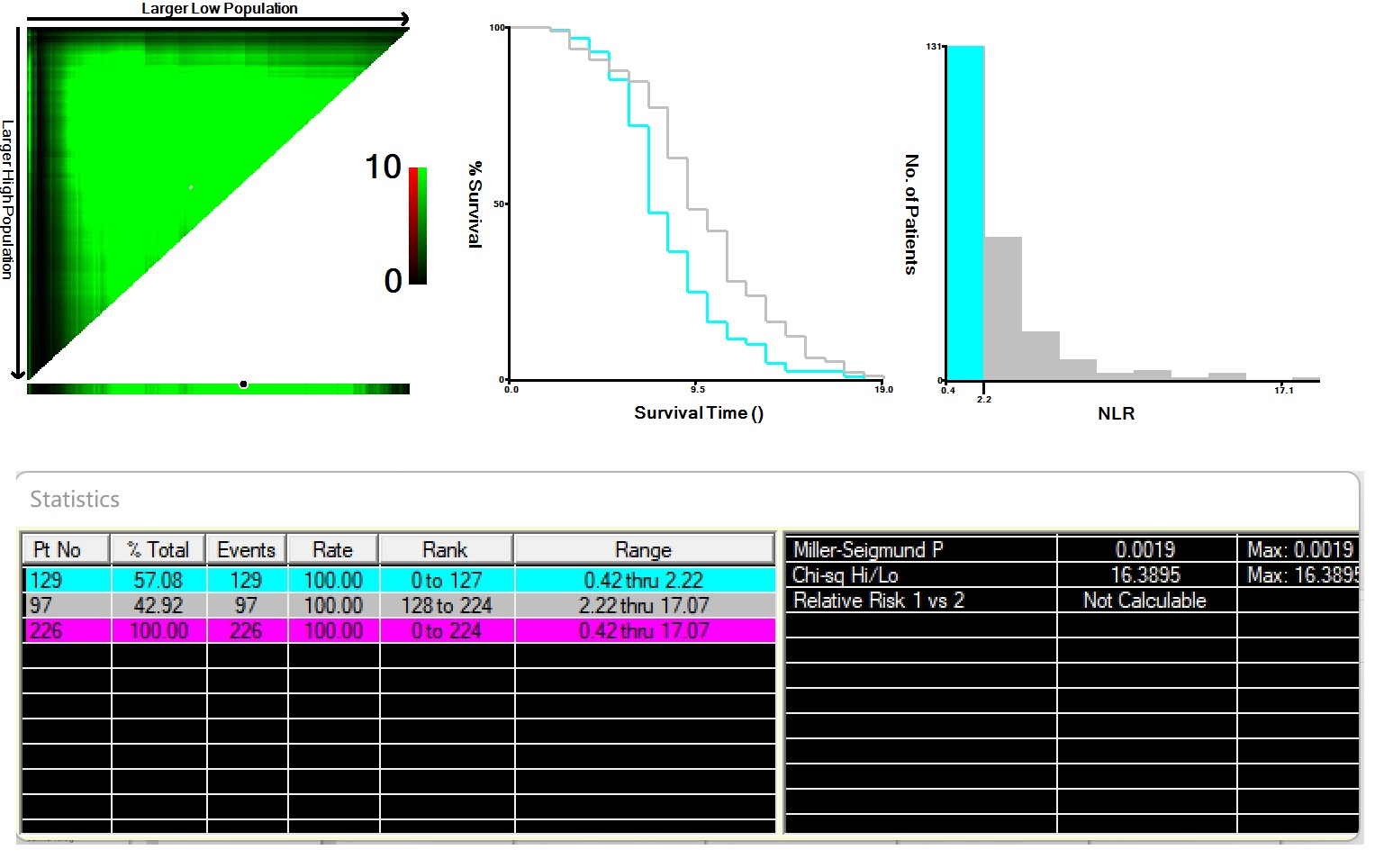

Supplement: Supplementary Materials — Supplementary Figure 1: the optimal cut-off value for NLR was calculated by X-tile software. [file 9576855.f1.jpg]
